# Endogenous Ouabain: An Old Cardiotonic Steroid as a New Biomarker of Heart Failure and a Predictor of Mortality after Cardiac Surgery

**DOI:** 10.1155/2015/714793

**Published:** 2015-11-01

**Authors:** Marco Simonini, Simona Pozzoli, Elena Bignami, Nunzia Casamassima, Elisabetta Messaggio, Chiara Lanzani, Elena Frati, Irene Maria Botticelli, Francesco Rotatori, Ottavio Alfieri, Alberto Zangrillo, Paolo Manunta

**Affiliations:** ^1^Genomics of Renal Diseases and Hypertension Department, “Vita-Salute” San Raffaele University, Chair of Nephrology, IRCCS San Raffaele Scientific Institute, 20132 Milan, Italy; ^2^Anesthesia and Intensive Care Department, “Vita-Salute” San Raffaele University, IRCCS San Raffaele Scientific Institute, 20132 Milan, Italy; ^3^SUNY Downstate Medical Center, State University of New York, Brooklyn, NY 11203, USA; ^4^Cardiac Surgery Department, “Vita-Salute” San Raffaele University, IRCCS San Raffaele Scientific Institute, 20132 Milan, Italy

## Abstract

Cardiovascular diseases remain the main cause of mortality and morbidity worldwide; primary prevention is a priority for physicians. Biomarkers are useful tools able to identify high-risk individuals, guide treatments, and determine prognosis. Our aim is to investigate Endogenous Ouabain (EO), an adrenal stress hormone with hemodynamic effects, as a valuable biomarker of heart failure. In a population of 845 patients undergoing elective cardiac surgery, we have investigated the relationships between EO and echocardiography parameters/plasmatic biomarker of cardiac function. EO was found to be correlated negatively with left ventricular EF (*p* = 0.001), positively with Cardiac End-Diastolic Diameter (*p* = 0.047), and positively with plasmatic NT-proBNP level (*p* = 0.02). Moreover, a different plasmatic EO level (both preoperative and postoperative) was found according to NYHA class (*p* = 0.013). All these results have been replicated on an independent cohort of patients (147 subjects from US). Finally, a higher EO level in the immediate postoperative time was indicative of a more severe cardiological condition and it was associated with increased perioperative mortality risk (*p* = 0.023 for 30-day morality). Our data suggest that preoperative and postoperative plasmatic EO level identifies patients with a more severe cardiovascular presentation at baseline. These patients have a higher risk of morbidity and mortality after cardiac surgery.

## 1. Introduction

Cardiovascular diseases are the leading cause of mortality and morbidity in the world [1]. Their primary prevention and secondary prevention are a priority for the health system and require multiple approaches to increase effectiveness [2]. Biomarkers are useful tools used to identify with greater accuracy high-risk individuals, establish a faster diagnosis, guide treatment, and determine prognosis [3].

Our research group deals with the role of Endogenous Ouabain (EO) as biomarker of clinical and subclinical cardiovascular disease.

Endogenous Ouabain (EO) is cardiac glycoside acting as an adrenal stress hormone with cardiological, hemodynamic, and renal effects. This hormone increases to picomolar (10^−12^) range in the plasma of hypertensive humans [4], after acute physical exercise [5], and in pregnancy [6]. EO is also known to be higher in patients with kidney failure [7], myocardial infarction [8], and congestive heart failure [9]. In addition to its hypertensive effects, EO even modifies cardiac function and modulates cellular proliferation and differentiation in heart [10], kidney [11, 12], and vascular smooth muscle [13]. Finally, it is also able to increase myogenic tone and reduce renal blood flow [7]. The primary site of Ouabain action is generally assumed to be the *α*-subunit of Na^+^K^+^-ATPase. Ouabain inhibits Na^+^K^+^-ATPase whit high affinity binding mainly to the *α*2 and *α*3 isoforms in vascular and brain tissues, respectively. This inhibition increases the Na^+^ concentration in the cytoplasm, which reduces the activity of the Na^+^/Ca^2+^ exchanger (NCX) and, consequently, increases the amount of Ca^2+^ available to activate contraction in tissues such as the heart. This produces a positive inotropic effect [14, 15]. A large number of experiments support the hypothesis that Na^+^K^+^-ATPase inhibition is not necessary for the inotropic effect of cardiac glycoside in the myocardium [16–20]. Recent studies have shown that the binding of Ouabain to Na^+^K^+^-ATPase elicits numerous additional changes in cell function. These include activation of intracellular signal transduction, activation of cytoplasmic Ca^2+^ oscillation, stimulation of endocytosis, and inhibition of endocytosis membrane traffic, as well as cell proliferation and adhesion [21–24]. Considering the important role of cardiac glycoside in cell signaling, growth, and apoptosis, it seems clear that these molecules represent potential biomarkers for acute and chronic kidney failure, heart failure, and cardiovascular remodeling as well as potential therapeutic targets.

### 1.1. EO and Heart Failure

The impact of an endogenous Na^+^K^+^-ATPase inhibitor on individuals with congestive heart failure could be considerable. First, the myocardial inotropic state is directly dependent on the function of the sodium potassium pump. Second, inhibition of the Na^+^K^+^-ATPase in the renal tubule may lead to natriuresis. Third, inhibition of the pump in the vasculature might maintain or increase blood pressure by causing vasoconstriction either directly [25] or by effects on sympathetic innervation [26]. These possible consequences of the actions of Na^+^K^+^-ATPase inhibitor explain the efficacy of cardiac glycosides in some patients and suggest that deficiency of EO might exacerbate congestive heart failure. On the other hand, the decreased cardiac output, fluid overload, and hypotension associated with congestive heart failure might increase adrenal release of EO. The relationship between Cardiotonic Steroids, cardiac geometry, and central hemodynamic parameters has been analyzed in several studies. Gottlieb et al. [27] found that although plasma EO did not exhibit graded increases with the progression of cardiac failure, EO levels were elevated in patients with severely impaired left ventricular (LV) performance (LV ejection fraction less than 21%); Manunta et al. [4] demonstrated that plasma EO positively correlated with systolic and diastolic blood pressure in a group of 110 normotensive subjects and 100 hypertensive subjects. These works demonstrated that EO levels correlated positively with LV mass index and LV end-diastolic volume only in hypertensive subjects. Later it was found that circulating EO levels in 92 hypertensive patients were positively correlated with mean BP and total peripheral resistance index, whereas LV end-diastolic volume index, stroke index, and cardiac index exhibited inverse correlations with this hormone. Plasmatic EO was found as independent predictor of total peripheral resistance index, cardiac index, and relative wall thickness. Moreover, the plasma EO was substantially higher in patients with eccentric remodeling compared with those subjects with normal LV geometry or concentric hypertrophy [28]. In another study performed in patients with LV dysfunction, the plasma EO was found to be elevated if compared with normal subjects [29]. Experimental data also indicate an association between elevated plasmatic Cardiotonic Steroids levels and cardiovascular remodeling. Moreover, sustained Ouabain infusion, which causes a 2-fold elevation of plasmatic Ouabain immunoreactivity, is sufficient to induce LV hypertrophy in normotensive rats [30].

### 1.2. Aim

The aim of this study is to evaluate if EO could be used as a valuable biomarker of heart failure. Moreover, we have studied the possible relationship between EO and clinical outcomes (as development of postsurgical complication and mortality rate) for those patients undergoing cardiac surgery.

## 2. Methods

### 2.1. Study Population

We enrolled more than 840 patients undergoing elective cardiac surgery (Coronary Artery Bypass Graft, valve surgery, Aortic Arch surgery, or a combination of previous interventions) at our hospital in the last five years (from December 2009 to December 2014). Cardiovascular risk factors, demographic data, clinical data (including past medical history), and medications were obtained from patient interview and chart review. We excluded patients with evidence of severe renal disease (as acute kidney injury (AKI) before surgery or End Stage Renal Disease (ESRD)), prior kidney or heart transplantation, or surgery performed in urgency. Participants with multiple surgeries were only enrolled in the study once. All participants provided written informed consent and institution research ethics board approved the study.

### 2.2. Validation Cohort

147 patients were enrolled in a prospective observational validation study conducted from January to April 2012 in the State University of New York, SUNY Downstate Medical Center (New York, US). Patients were admitted to the cardiology service or in the Intensive Care Unit (ICU) of the Cardiology Department. We measured EO levels in patients with heart failure, acute coronary disease, atrial and ventricular arrhythmias, and systemic or pulmonary hypertension admitted to the cardiology service. Written consent was obtained from the participating subjects (see Supplementary Material for details, available online at http://dx.doi.org/10.1155/2015/714793).

### 2.3. Sample Collection

We collected urine and plasma specimens preoperatively and daily until ICU discharge. The first postoperative samples were collected 24 h after cardiac surgery was performed. In addition to routine preoperative assessments, blood samples were obtained for plasma EO determinations within 24 hours from admission to the clinic and for the first 24 hours postoperatively. Samples were stored at −80°C until analysis.

### 2.4. Determination of EO

EO was extracted from plasma and measured by using a specific radioimmunoassay (RIA) in accordance with those methods previously published [31, 32].

Plasma samples are preextracted with methanol, dried with speed vacuum, and reconstituted with 0.1% trifluoroacetic acid. Preconditioned C18 Bond Elut columns (Varian, Inc., Palo Alto, California, US) are used for sample extraction. Following several water washes and one wash with 2.5% acetonitrile, Endogenous Ouabain is eluted with 25% acetonitrile. These steps are critical for two main reasons: first, the recovery of EO from the column can be variable if the preconditioning is not correct, and second the 2.5% acetonitrile wash reduces the highly polar material present in plasma extracts. Rabbit polyclonal antiouabain antisera of high titer (prepared by two different Institutes) were used in the radioimmunoassay. These antiouabain antisera possessed panels of cross-reactivity very specific for EO and similar aglycones. The cross-reactivity of the two antisera with the purified human plasma Endogenous Ouabain differed slightly from one to each other, while always remaining within experimental error (Hamlyn, unpublished observation). The possibility that the antisera used in our radioimmunoassay may recognize the human placental inhibitor of Na^+^-K^+^-ATPase, whose suggested structure resembles that of a bufadienolide, is very unlikely because the two Ouabain antisera we used show very weak cross-reactivity with the bufadienolides (<1%). Moreover, the sample extraction process we employ precludes the presence of bufadienolides in the Endogenous Ouabain assay because they are not sufficiently polar to be eluted by 25% acetonitrile under our conditions. Another variable is the method used to separate bound from free-labeled Ouabain. For the Milan Endogenous Ouabain radioimmunoassay system, the intra-assay and interassay coefficient of variation were approximately 5 and 9%, respectively, and remain remarkably steady over the years. When all aspects of the radioimmunoassay method are respected, all tissues from rats and humans including plasma contain measurable and reproducible amounts of EO by radioimmunoassay and ATPase assay, both before and after HPLC fractionation. An investigator blinded to the plasma biomarker concentration collected data from patient chart notes and the computerized data system.

### 2.5. Statistical Analyses

Continuous data are expressed as means ± standard deviation. Dichotomous variables are presented as percentages. Median and interquartile ranges (IQR) are presented for nonparametric variables. Reflecting the nonnormal distribution of EO among the population [30], we used logarithmic transformation for the statistical analysis or nonparametric tests when appropriate. ANOVA or Kruskal-Wallis and median tests were used to compare continuous variables among NYHA or left ventricular EF class, whereas Chi-square analysis or Fisher's test was used to compare discrete variables. The Mann-Whitney test was used to compare EO between different groups. Logistic Regression was used to study the effect of different variables on mortality rate. ROC was used to examine the predicting power of different variables on mortality rate; AUC (i.e., C-index) was calculated from the ROC curve. A statistically derived value, based on the Youden index [33], maximizing the sum of the sensitivity and specificity, was used to define the optimal cut-off value. A two-sided *p* value of <0.05 was considered to indicate statistical significance. All analyses were performed with SPSS 22.0 software (IBM, Inc., Armonk, NY, USA).

## 3. Results

The study population was composed of 845 patients (34.4% females and 65.6% males; details in Table 1). Postoperative AKI (according to AKIN criteria [34]) was observed in 197 patients (23.3%). Total in-hospital mortality was 1.7% (14 patients) for cardiovascular complications after surgery; 30-day mortality was 1.3% (11 patients). All deceased patients developed AKI before exitus.

### 3.1. EO as Biomarker of Cardiac Failure

We have focused our attention on the relationships among preoperative clinical status of the patient, specific echocardiographic parameters of cardiac function, and plasmatic markers used in common clinical practice. In particular, as it is known from the literature [35–38], in our population a significant negative correlation between left ventricular ejection fraction (LVEF) with Cardiac End-Diastolic Diameter (*p* < 0.001, *r* = 0.487, Pearson Correlation) and with preoperative value of NT-proBNP (Pearson Correlation *r* = 0.569; *p* < 0.001) was confirmed.

**Figure 1:**
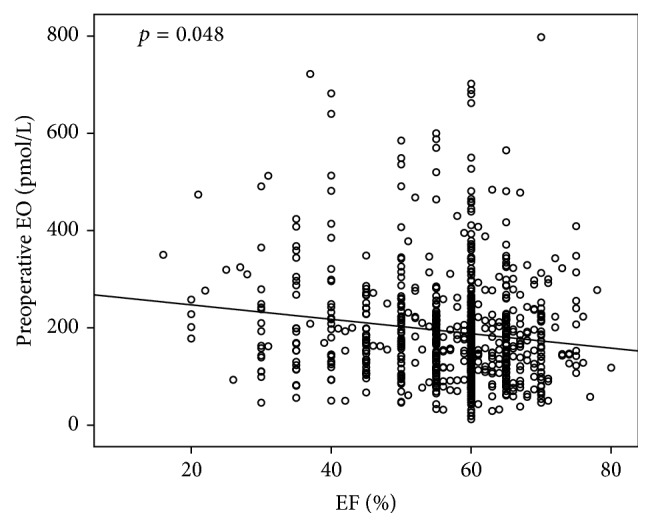
Correlation between baseline (preoperative) EO level and cardiac left ventricular ejection fraction (LVEF). Patients with higher Endogenous Ouabain baseline levels are those with lower LVEF (Pearson Correlation with logarithmic EO *r* = 0.135; *p* = 0.001 (0.048 after correction for sex, age, BMI, preoperative GFR, and clinical presentation expressed as EuroSCORE)).

**Figure 2:**
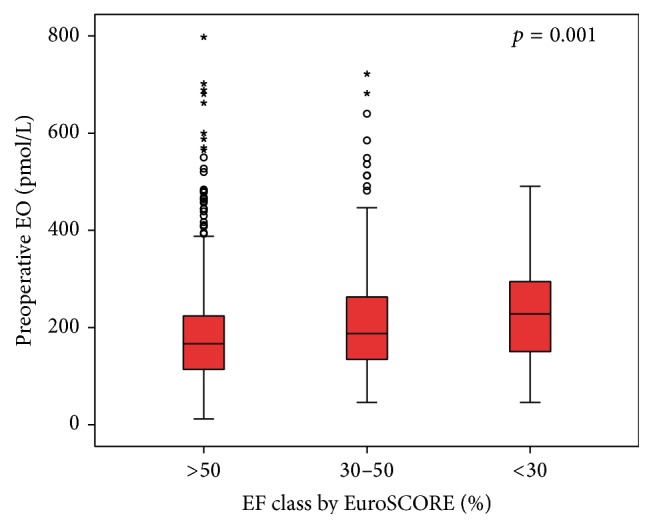
EO preoperative level according to EuroSCORE EF classification (three classes: EF < 30%; EF 30–50%; EF > 50%; Kruskal-Wallis *p* = 0.001).

We found a correlation between baseline (preoperative) EO level and cardiac ejection fraction. Patients with a higher baseline Endogenous Ouabain are those with lower left ventricular ejection fraction (Pearson Correlation with logarithmic EO *r* = 0.135; *p* = 0.001 (0.048 after correction for covariates); Figure 1). Results remain significant even after LVEF was recorded according to EuroSCORE [39] classification (three classes: EF < 30%; EF 30–50%; EF > 50%; Kruskal-Wallis *p* = 0.001 (0.013 after correction for covariates); Figure 2). Moreover a positive correlation between preoperative level of Endogenous Ouabain and Cardiac End-Diastolic Diameter was also observed (Pearson Correlation with logarithmic EO: *r* = 0.147; *p* = 0.047 (0.05 after correction for covariates)). Furthermore, a positive correlation was observed also between the plasmatic values of EO and NT-proBNP (Pearson Correlation with logarithmic EO: *r* = 0.321; *p* = 0.02 (0.021 after correction for covariates)). Statistical adjustment was made for sex, age, BMI, preoperative GFR, and clinical presentation (summarized by EuroSCORE preoperative value).

**Figure 3:**
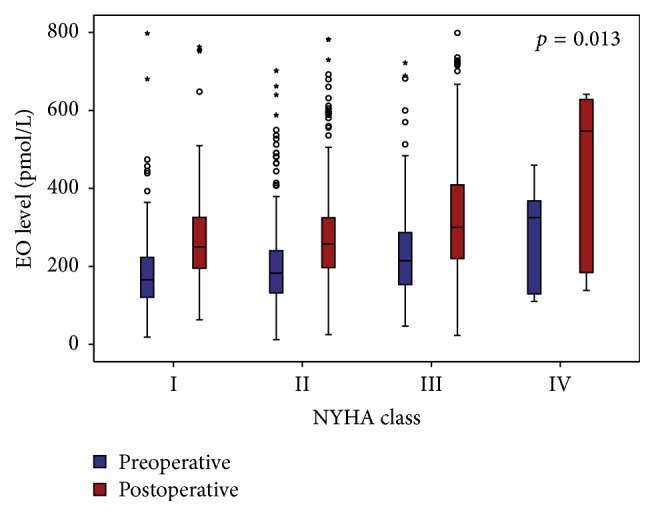
EO levels (blue = preoperative; red = postoperative) according to each NYHA class (*p* = 0.013 after correction for sex, age, BMI, preoperative GFR, EuroSCORE, and baseline level of EO).

Finally, those patients with a more severe heart failure index, expressed as NYHA class, have a higher baseline EO plasmatic level (*p* = 0.047). According to each NYHA class, mean (±SD) EO preoperative level (expressed in pmol/L) was as follows: 179.84 ± 107.58 for class I; 192.07 ± 107.45 for class II; 209.08 ± 125.67 for class III; and 247.98 ± 133.52 for class IV. More interesting, we observed the same trend, but with a stronger evidence, for postoperative EO level (mean ± SD, resp.: 272.81 ± 127.59 versus 276.89 ± 126.50 versus 333.62 ± 164.78 versus 427.79 ± 246.65 pmol/L) according to NYHA class (Kruskal-Wallis *p* = 0.0001; Figure 3). Correlation between postoperative EO and NYHA class was corrected for clinical variables (see above) and also for preoperative level of EO (*p* = 0.013).

As replication we performed the same analysis on a different population of 147 subjects from SUNY Downstate Medical Center (New York, US). Despite the small sample size, we confirmed the main results observed on the Italian cohort. In particular, we observed a negative correlation between EO preoperative level and LVEF (*p* = 0.008) and a positive correlation EO-BNP (*p* = 0.05). See supplementary data for details.

### 3.2. EO as Biomarker of Cardiac Stress

In order to support the hypothesis that EO was secreted during hemodynamics stress [40], we investigated the change in EO plasmatic level according to different kind of cardiac pathology or surgical technique.

A higher circulating preoperative level of EO was found in patients undergoing Coronary Artery Bypass Graft (CABG) intervention. This difference is most evident considering the values of patients with simple valvular disease eligible for surgical repair (ANOVA *p* = 0.01). In the same way, those patients undergoing a more complex surgical procedure (e.g., patient with surgery on Aorta Arch or undergoing combined intervention) reached a higher level of postoperative EO plasmatic concentration (*p* = 0.009). See Table 2 for details.

Moreover, when we looked to surgical technique, a greater plasmatic EO levels change was observed (meant as postoperative/preoperative EO variation (ΔEO)), in those patients undergoing cardiopulmonary bypass (CPB). ΔEO was, respectively, 43.73 ± 134.65 pmol/L (NO-CPB) versus 87.62 ± 131.56 pmol/L (CBP), ANOVA *p* = 0.007. This result was even more significant when we have restricted the analysis only to those patients undergoing CABG as surgical intervention (NO-CBP (*n* = 55): 26.89 ± 124.05 versus CBP (*n* = 37): 97.90 ± 80.87 pmol/L; ANOVA *p* = 0.003).

### 3.3. EO as Predictor of Postoperative Morbidity and Mortality

In our previous studies we have already demonstrated that baseline values of Endogenous Ouabain correlate with the development of AKI after cardiac surgery [41, 42]. Also in this subset of patients we confirmed the same predictive power. Indeed, patients with higher EO baseline values have higher chance to develop mild to severe AKI after cardiac surgery (Logistic Regression for AKI *p* < 0.0001). All data were corrected for clinical risk model for AKI [42]. This result was greatly expected because the population studied in this work is largely superimposed on those previously published and represents an expansion of the same population. In this work we tried to find any correlation between postoperative EO levels and the development of postoperative renal failure. Unfortunately, a weaker correlation with AKI of postoperative EO if compared with preoperative EO was observed (Logistic Regression for AKI *p* = 0.017); moreover, in a risk model including clinical variables and both EO levels (pre- and postoperative), only basal EO was related with AKI development.

In our sample the mortality was approximately 1.4%; this data was concordant with those reported in the literature [43, 44]. In particular, deaths by cardiovascular events were 14 (indeed patients who died from septic events or surgical complications were excluded from this work).

In our population mortality rate has been associated with baseline eGFR, previous cardiac surgery, and preoperative NYHA class (see Table 3). Moreover, all deceased patients developed AKI before “exitus” occurred (probably as a consequence of the development of heart failure). These data are perfectly in agreement with the literature [45]. All these variables are summarized by EuroSCORE (European System for Cardiac Operative Risk Evaluation) value [39]. EuroSCORE is able to predict perioperative (30 days) mortality [46] for patients undergoing cardiac surgery. This score is very well codified and widely accepted: indeed the use of this score to predict mortality risk is codified by International Guidelines [44].

We confirmed the excellent existing correlation between EuroSCORE and mortality rate. A greater EuroSCORE preoperative value was observed in those patients who died after surgery if compared to those who have survived (resp., 3.89 ± 4.04 versus 11.36 ± 14.68; ANOVA *p* < 0.0001). Moreover, Logistic Regression for total in-hospital mortality was strictly significant (Logistic Regression *p* = 0.003; Table 3).

Any significant relationship between preoperative value of EO and mortality rate was not observed. Furthermore, also the change in EO plasmatic levels was not correlated with total in-hospital mortality after correction for EuroSCORE.

We found that only the postoperative value of Endogenous Ouabain was strongly related with mortality rate after correction for baseline EuroSCORE value: in particular, those patients with a higher level of postoperative EO have a higher mortality risk after cardiac surgery (Logistic Regression *p* = 0.046). To better understand the impact of postoperative EO on mortality risk we performed Receiver Operating Characteristic (ROC) curve (AUC 0.68 ± 0.08; *p* = 0.035) to allow Youden's index calculation [33]: we identified 363 pmol/L as critical EO level. Those patients with plasmatic EO concentration after cardiac surgery > 363 pmol/L had a mortality risk 3.5 times higher if compared to other patients.

It is also well know that NT-proBNP is a good predictor of postoperative mortality [47–49]. Even in the studied population pre- and postoperative levels of NT-proBNP were higher in deceased patients if compared to those who have survived (Kruskal-Wallis *p* = 0.036 and *p* = 0.039 for preoperative and postoperative NT-proBNP values, resp.). However, the predictive power of this biomarker of heart failure (expressed as Logistic Regression for mortality) was found to be of borderline significance and, surprisingly, less effective if compared to EO (Logistic Regression for NT-proBNP: *p* = 0.085 and *p* = 0.052 for preoperative and postoperative NT-proBNP values, resp.). After correction for EuroSCORE no association between NT-proBNP and mortality rate was observed. In our opinion this unexpected result could be explained by the small number of recorded events that could mask the real “potential” of NT-proBNP.

**Figure 4:**
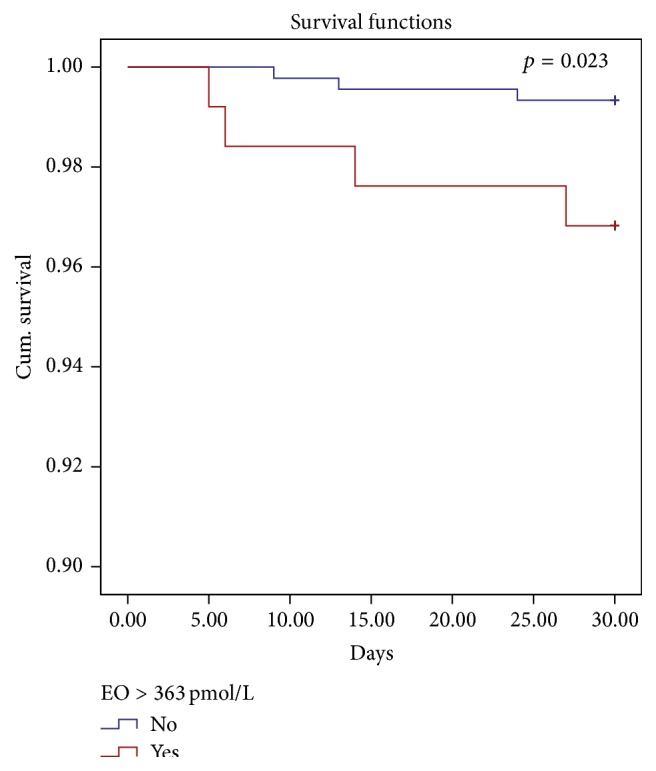
30-day survival rate for patients with high (>363 pmol/L, red line) postoperative EO level (Cox regression corrected for EuroSCORE *p* = 0.023).

Finally, because EuroSCORE was codified for 30-day mortality prediction [46], we performed a survival analysis at 30 days after cardiac surgery. In this case mortality was 1.3% (11 patients). Also in this analysis postoperative EO was confirmed to be associated with 30-day mortality (Cox regression *p* = 0.023 after correction for EuroSCORE; Figure 4).

## 4. Conclusion

This work tried to investigate the relationship among EO and several clinical and biological heart-related variables in order to assess EO as a new and valuable biomarker for heart failure. Moreover, we investigated the role of EO pre- and postoperative levels on cardiovascular mortality after cardiac surgery.

The main results were the evidence of correlations between EO and several echocardiographic parameters of cardiac function. EO correlates negatively with LVEF (*p* = 0.001) and positively with Cardiac End-Diastolic Diameter (*p* = 0.047). Moreover, an increase in plasma EO concentration is associated with an analogue increase of plasmatic NT-proBNP (*p* = 0.02), a well-known and well-accepted biomarker of cardiac failure. All these results are of special interest because they are clinically related one to each other: a patient with heart failure will show reduced EF, increased BNP, and a concomitant rise in EO level. The evidence of different plasmatic EO level according to NYHA class is not a simple consequence of the previous results; it is another demonstration of the close relationship between high levels of EO and impaired cardiac function. Furthermore, all these results have been replicated on an independent control cohort of patients (147 subjects from Cardiology Unit of SUNY Downstate Medical Center). This makes such results even more significant.

The second evidence of this paper is the further demonstration that EO acts as an acute stress hormone. Patients undergoing a more complex surgical procedure (as a combined intervention or a procedure that involves Aortic Arch) reached a higher level of postoperative EO. Moreover patients undergoing CPB (a surgical technique in which cardiorenal system is exposed to an intense hemodynamic stress with a significant release of proinflammatory cytokines [50]) have a greater increase in postoperative EO plasmatic level (*p* = 0.009).

Finally, we have shown that high levels of EO in the immediate postoperative time are indicative of a more severe heart condition and how they are associated with increased perioperative mortality (*p* = 0.023 for 30-day morality).

All these data, taken together, bring to reconsider EO no longer as a simple natriuretic hormone. These evidences raise awareness that EO is a real cardiac stress hormone and that could be an indicator of the presence of subclinical cardiovascular damage. It is yet to be investigated whether this subclinical damage is mainly important for the cardiac, vascular, or kidney district and what is the first organ to be involved. Furthermore, our data suggest that those patients with a more severe heart failure (and, as consequence, with a reduction in blood pressure (BP) levels) may have an increase in EO plasmatic levels in response to the hypotensive stimulus. This could occur in attempt to restore BP values to physiological levels. These findings are in agreement with other previous observations on the effects of extracorporeal circulation (ECC) on EO values. Indeed, Bignami et al. [40] have shown how EO markedly and rapidly increases after significant reduction of BP values induced by the anesthesia.

Our data indicate that EO acts as a biomarker of individual cardiovascular condition. Presence of higher preoperative and postoperative plasmatic EO level identifies patients with a higher risk of morbidity and mortality after surgery. Those patients may benefit from inhibition of EO action. Indeed an inhibitor of EO (rostafuroxin) has been recently developed [51] and might help to minimize perioperative mortality. Moreover, the theoretical possibility of a pharmacological intervention able to change EO levels makes the study of this hormone of particular interest. Actually, in contrast to traditional biomarkers of heart failure (such as NT-proBNP), which are used only for diagnostic, EO could be used with a double purpose: diagnostic (preoperative) and therapeutic (postoperative). But to reach this futuristic goal we need further investigations to understand the real impact of EO on the development/progression of cardiovascular damage and its role of “link” among HF, AKI, and mortality.

## 5. Limitation

There are several limitations in this study that will require further investigation.

The most important one seems to be the small number of events (death) on which we have conducted the analysis. Actually the mortality rate of our population is comparable with the literature. In addition, our hospital is considered a “Center of Excellence” in Italy for the cardiac surgery, especially for the great experience of our surgical team; this further reduces the number of recorded events. Nonetheless, we believe that the results presented should be considered truthful for three reasons. First, all surgical operations were conducted by the same surgical team. Moreover, all preoperative echocardiographic studies were conducted by the same team of two people, minimizing the intraoperator variability. These peculiarities make this population highly homogeneous and reduce confounding variables. This allows us to study in detail the pathophysiological mechanisms underlying the development/worsening of cardiovascular diseases and to obtain interesting results even with a small number of reported events. Second, to further reduce confounding factors, we have chosen to include in the study only surgical interventions performed electively, excluding emergencies and patients with too severe comorbidities (such as ESRD). Third, in order to be sure of investigating the effect of EO on cardiovascular mortality, we excluded “a priori” from the analysis all patients who died from other causes (such as septic complications or issues related to surgical procedure).

Another point of discussion could be the choice not to correct “EO-mortality” association for presence of postoperative AKI. In fact, as is well known, there is a very strong association between postoperative mortality and the onset of renal damage. This is also evident in our population (see Section 3 and Table 3). However, considering that all the deceased patients developed kidney damage, AKI has a statistical power so strong to be able to cover all other variables under examination (including EuroSCORE, EF, and other clinical characteristics well known in the literature as being associated with perioperative mortality). For this reason, we have chosen excluding AKI from the analysis. In addition, in our opinion, kidney damage is secondary to the development of heart failure (triggering factor of reported mortality events) and the final aim of this study is to investigate the relationship between EO and heart failure (also because the relationship between EO and AKI has already been thoroughly demonstrated [41, 42, 52]).

Finally, for the statistical analysis was used EuroSCORE instead of EuroSCORE II (a recent evolution EuroSCORE). This was decided for two reasons. First, patient enrollment was started before the publication of this new score (2009 versus 2011) and not for all patients could the new EuroSCORE II be calculated (especially for older ones). So we chose to maintain the old score in order to preserve sample size. Second, in the most recent guidelines [44] EuroSCORE is still the reference model for calculation of perioperative mortality.

## Supplementary Material

In a independent cohort of 147 subject (from Cardiology Unit of SUNY Downstate Medical Center, New York) we obtained results similar to those presented in the main article on the relationship between EO and heart failure (a negative correlation was observed between EO and Left Ventricular Ejection Fraction (LVEF); a positive correlation was found between EO and Left Ventricular mass standardized to body surface area (LVMI); a positive correlation was found between EO and NT-proBNP).

## Figures and Tables

**Table 1:** Characteristics of population.

Population characteristics (845 subjects)	
Anthropometric and preoperative parameters	

Gender (f/m, %)	34.4/65.6
Age (years)	62.40 ± 13.14
BMI (kg/m^2^)	25.28 ± 4.11
Plasma creatinine (mg/dL)	0.90 ± 0.23
eGFR (mL/m 1.73 m^2^)	80.25 ± 20.47
Hypertension (%)	55.4
Diabetes (%)	14.5
Peripheral vascular disease (%)	17.7
Chronic obstructive pulmonary disease (%)	11.5
EuroSCORE	4.03 ± 4.54
CLIN-RISK^#^	9.38 ± 4.08
Plasma EO (pmol/L)^*∗*^	174 [118–241]
	204.62 ± 145.96
NT-ProBNP (pg/mL)	680.72 ± 910.53

Cardiological characteristics	

Left ventricular ejection fraction (%)	56.98 ± 10.27
Class LVEF (%)	
<30%	3.3
30–50%	19.9
>50%	76.8
Interventricular septum (mm)	11.31 ± 2.12
Cardiac End-Diastolic Diameter (mm)	53.86 ± 7.95
NYHA classification (%)	
I	22.1
II	55.9
III	21.0
IV	1.1

Surgical characteristics	

Surgery type (%)	
Valve repair (VR)	49.5
Isolated coronary bypass (CABG)	16.3
CABG + VR	18.5
Aortic Arch surgery	13.3
Other	2.2
Reoperation cardiac surgery (%)	10.7
Combined surgery (%)	18.6
Cardiopulmonary bypass used (%)	88.9
Cardiopulmonary bypass duration (min)	68.85 ± 30.04

Postoperative time	

Plasma creatinine (mg/dL)	1.20 ± 0.63
Plasma EO (pmol/L)^*∗*^	267 [200–357]
	311.55 ± 200.70
ΔPlasma EO (pmol/L)	91.51 ± 195.52

Outcomes	

AKI (%)	
AKIN Stage I	23.3
AKIN Stage II	9.3
AKIN Stage II	2.3
In-hospital mortality (*n*, %)	14 (1.7%)

Dichotomy variables: expressed as % (positive).

Parametric variables: expressed as mean ± s.d.

Nonparametric variables (*∗*): expressed as median (25–75 percentile) and mean ± s.d.

^#^Clinical risk model for postoperative severe AKI (based on gender, age, LVEF, hypertension, diabetes, renal function, reintervention, and surgery type (see [41, 42])).

**Table 2:** EO levels according to different surgical intervention.

Time		Number of patients	Mean (pmol/L)	SE	ANOVA *p* value^#^	Multiple comparison *p* value^#^
Preoperative	VR	401	183.74	5.57	0.01	
	CABG	136	206.17	8.52		0.005 versus VR
	Complex	280	201.01	7.002		0.07 versus VR

Postoperative	VR		270.84	7.91	0.009	
	CABG		278.93	9.64		
	Complex		313.34	10.91		0.003 versus VR

ΔEO	VR		79.54	7.62	0.033	
	CABG		56.08	11.10		
	Complex		96.98	9.94		0.01 versus CABG

VR: valve repair; CABG: Coronary Artery Bypass Graft; complex: combined surgery + Aortic Arch surgery.

^#^ *p* value for ANOVA (with EO transformed as logarithmic).

ΔEO: postoperative − preoperative.

**Table 3:** Logistic Regression with mortality.

	Variables	*p* value^#^	*p* value^*∗*^	Exp(*B*)^*∗*^	CI (95%)^*∗*^
Reference	EuroSCORE	0.001	—	1.10	1.03–1.18

Clinical characteristics	Age	0.004	0.039	1.08	1.00–1.16
	LVEF	n.s.	n.s.		
	NYHA	0.027	n.s.		
	Basal creatinine	0.006	n.s.		
	Hypertension	n.s.	n.s.		
	Diabetes	n.s.	n.s.		

Surgery type	REDO	0.001	0.011	5.02	1.45–17.33
	Complex	0.023	0.04	2.17	1.04–4.56
	EEC	n.s.	n.s.		

Complications	AKI	<0.001	<0.001	53.24	10.99–257.87

EO	Preoperative EO	n.s.	n.s.		
	Postoperative EO	0.025	0.046	1.04	1.00–1.07
	Postoperative EO > 363 pmol/L	0.04	0.049	3.58	1.03–12.78

^#^ *p* value for Logistic Regression (not correct); ^*∗*^ *p* value and Exp(*B*) for Logistic Regression after correction for EuroSCORE.

n.s: not significant; Exp(*B*): expected beta for Logistic Regression; LVEF: left ventricular ejection fraction; REDO: reintervention; complex: combined surgery or Aortic Arch surgery; EEC: extracorporeal circulation; AKI: acute kidney injury (by AKIN criteria; see [34]).

## Abbreviations

Δ: Variation from baseline
AKI: Acute kidney injury
BNP: Brain natriuretic peptide
CABG: Coronary Artery Bypass Graft
CPB: Cardiopulmonary bypass
eGFR: Estimated glomerular filtration rate
EO: Endogenous Ouabain
EF: Ejection fraction
ECC: Extracorporeal circuit circulation
EDD: Cardiac End-Diastolic Diameter
ESRD: End Stage Renal Disease
HF: Heart Failure
LV: Left ventricular
NT-proBNP: N-terminal prohormone of brain natriuretic peptide.
