# Supplementary material for: Endogenous Ouabain: An Old Cardiotonic Steroid as a New Biomarker of Heart Failure and a Predictor of Mortality after Cardiac Surgery
Source: Biomed Res Int. 2015 Nov 1;2015:714793. doi: 10.1155/2015/714793 (PMC4644558; doi:10.1155/2015/714793)
Supplement: Supplementary file 1 — In a independent cohort of 147 subject (from Cardiology Unit of SUNY Downstate Medical Center, New York) we obtained results similar to those presented in the main article on the relationship between EO and heart failure (a negative correlation was observed between EO and Left Ventricular Ejection Fraction (LVEF); a positive correlation was found between EO and Left Ventricular mass standardized to body surface area (LVMI); a positive correlation was found between EO and NT-proBNP). [file 714793.f1.pdf]

ENDOGENOUS OUABAIN: AN OLD CARDIOTONIC STEROID AS A NEW BIOMARKER OF HEART FAILURE AND A PREDICTOR OF MORTALITY AFTER CARDIAC SURGERY

Supplementary data

METHODS: 147 patients (126 African American (AA) and 21 Hispanic or Caucasian (Other)) were enrolled in a prospective observational validation study conducted from January to April 2012 at SUNY Downstate Medical Center (New York, US). Patients were admitted to the Cardiology Service or in the Intensive Care Unit of the Cardiology Department. We measured EO levels in patients with heart failure, acute coronary disease, atrial and ventricular arrhythmias and systemic or pulmonary hypertension admitted to the cardiology service. Written consent was obtained from the participating subjects. Blood samples were collected and processed as in the main article (see methods). During recovery time Color Doppler Echocardiography was performed to each patient and clinical data were recorded.

RESULTS: As table 1 shows, there was not significant difference between the two ethnical groups in both clinical or echocardiographic parameters. Moreover, there was no any significant difference in mean Endogenous Ouabain (EO) between the two groups: AA mean (± SD) EO was 249,35 ± 144,03 pmol/L vs 268,54 ± 125,89 pmol/L for "Other" group.
As already known from current literature [1], Endogenous Ouabain levels correlate with renal insufficiency. We found that patients with a plasmatic creatinine over 1.5 mg/dL had higher Ouabain levels if compared to patients with serum creatinine within normal laboratory range (mean (± SD) EO (pmol/L) 287,08 ± 156,59 vs 234,48 ± 130,51; Mann-Whitney p=0.046).

Similarly to the results of the main article, we found also in this subset of patients a relationship between EO and heart failure. In particular, a negative correlation was observed between EO and Left Ventricular Ejection Fraction (LVEF): Pearson Correlation (with Logarithmic EO) r-0.224; p=0.008 (Figure 1). Furthermore, a moderate positive correlation was found between EO and Left Ventricular mass standardized to body surface area (LVMI): Pearson Correlation (with Logarithmic EO) r=0.17; p=0.05. Finally, we also replicated the relationship between EO and NT-proBNP (r=0.18, p=0.05; figure 2).

If we consider only African American patients, main results remain significant. Endogenous Ouabain levels still correlate positively with reduced kidney function (p=0.034), negatively with LVEF (p=0.035), positively with NT-proBNP (p=0.05) and positively with LVMI (p=0.05).

In these population we found also a positive association between history of stroke (as previous Transient Ischemic Attack (TIA) or Stroke) with elevated EO levels. Over 147 patients, 13 (8.8%) had a positive anamnesis for stroke; for these subjects mean Endogenous Ouabain was 359.54 ± 173.12 pmol/L vs 241 ± 134.00 pmol/L for patients without any history of neurological events (Mann-Whitney p=0.006).

CONCLUSION: Our findings confirm and extend what has been reported in the main article: Endogenous Ouabain is increased in heart failure conditions. According to these findings, this hormone could be potentially used as a novel biomarker of cardiovascular injury as it seems to be for kidney failure. Moreover, in these specific population, with a predominance of AA subjects, EO correlates with LV mass standardized to body surface area (LVMI), a well-known predictor of morbidity and mortality in patients with cardiovascular disease.

Table 1: Population characteristics by ethnicity groups.

	African American (AA)	Other ethnicity (Other)	p value
N° of Subject	127	21	-
Age (years)	66 ± 13	66.26 ± 13	ns
Sex (M/F)	66/61	17/4	ns
BMI (Kg/m^2)	29 ± 7	29 ± 7	ns
Heart rate (bpm)	76 ± 16	77 ± 14	ns
SBP (mmHg)	126 ± 21	117 ± 18	0.071
DBP (mmHg)	72 ± 11	67 ± 14	0.062
Creatinine (mg/dl)	1.8 ± 1.8	1.4 ± 0.8	ns
eGFR (ml/min)	63 ± 30	66 ± 29	ns
BUN (mg/dl)	27 ± 18	25 ± 13	ns
NT-pro BNP (pg/ml)	673 ± 800	725 ± 994	ns
Hb (g/dl)	12.1 ± 1.9	12.5 ± 2.2	ns
LVEF (%)	39 ± 18	34 ± 16	ns
Lvids (mm)	43 ± 14	45 ± 14	ns
LA dim (cm)	4.3 ± 0.75	4.5 ± 0.7	ns
EDD (cm)	5.4 ± 1.1	5.6 ± 1.2	ns

Parametric variables = expressed as mean ± s.d.
BMI: body mass index; SBP: systolic blood pressure; DBP: diastolic blood pressure; BUN: blood urea nitrogen; BNP: brain natriuretic peptide; Hb: hemoglobin; LVEF: Left Ventricular Ejection Fraction; Lvids: Left Ventricular Internal Dimensions; LA dim: Left Atrium Dimensions; EDD: end diastolic diameters.

Figure 1: Correlation between EO (at baseline) and Left Ventricular Ejection Fraction (LVEF).

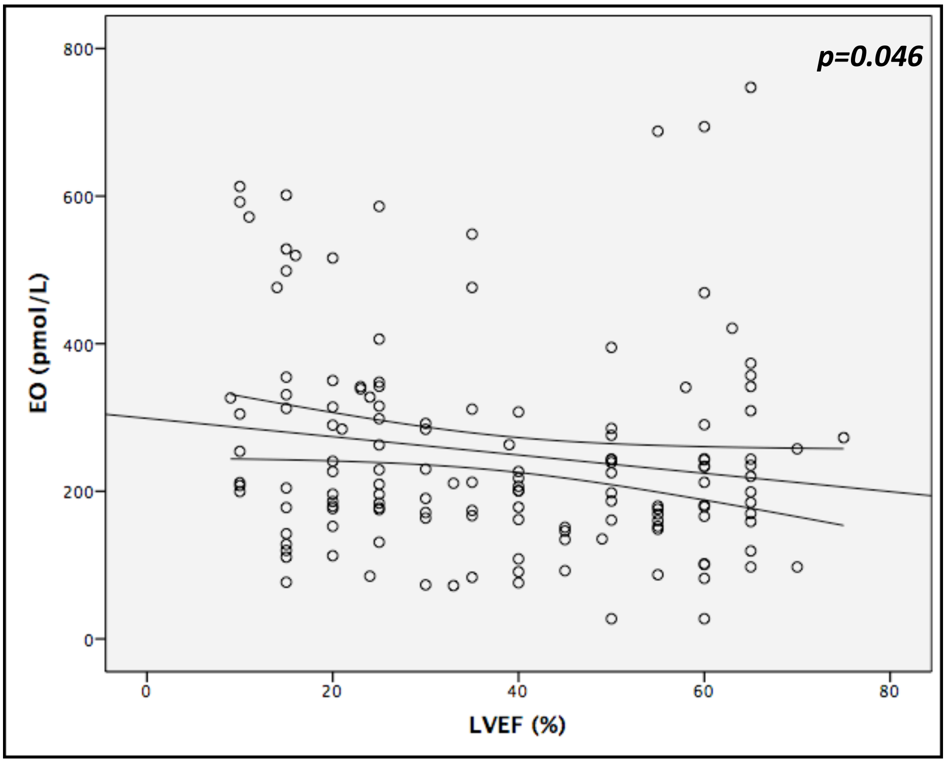

Figure 2: Correlation between EO (at baseline) and NT-proBNP. A) with raw numbers; B) after Logarithmic transformation.

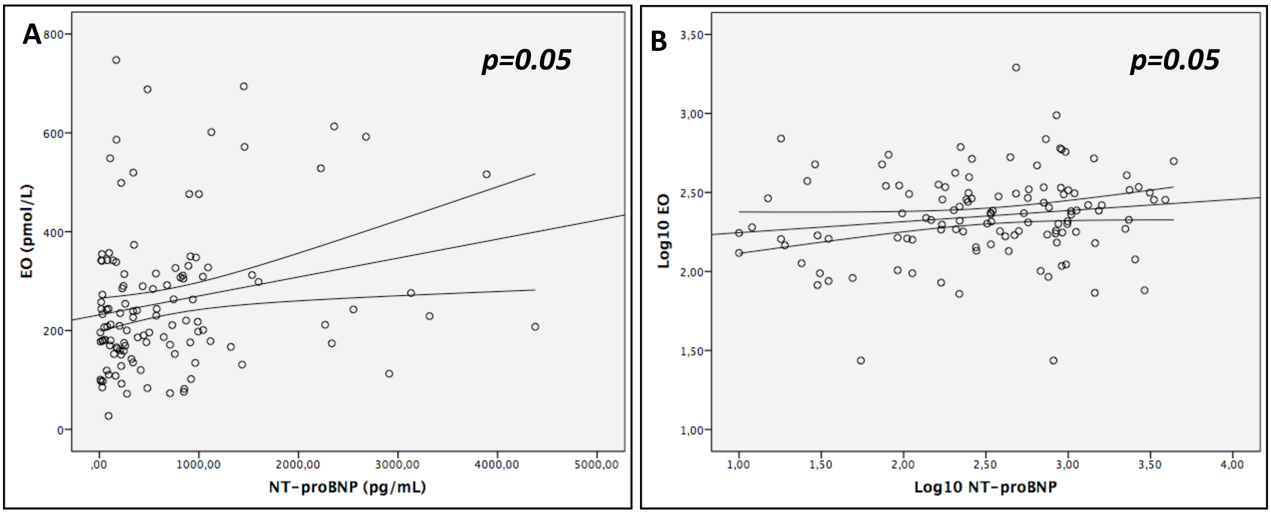

REFERENCE

[1] Hamlyn JM, Manunta P. Endogenous Cardiotonic Steroids in Kidney Failure: A Review and an Hypothesis. Advances in Chronic Kidney Disease 2015;22:232–44. doi:10.1053/j.ackd.2014.12.005.
